# Collective Chemotaxis Requires Contact-Dependent Cell Polarity

**DOI:** 10.1016/j.devcel.2010.06.012

**Published:** 2010-07-20

**Authors:** Eric Theveneau, Lorena Marchant, Sei Kuriyama, Mazhar Gull, Barbara Moepps, Maddy Parsons, Roberto Mayor

**Affiliations:** 1 Department of Cell and Developmental Biology, University College London, London WC1E 6BT, UK; 2 Institute of Pharmacology and Toxicology, University of Ulm, 89069 Ulm, Germany; 3 Randall Division of Cell and Molecular Biophysics, King's College London, London WC2R 2LS, UK

**Keywords:** SIGNALING, CELLBIO, PROTEINS

## Abstract

Directional collective migration is now a widely recognized mode of migration during embryogenesis and cancer. However, how a cluster of cells responds to chemoattractants is not fully understood. Neural crest cells are among the most motile cells in the embryo, and their behavior has been likened to malignant invasion. Here, we show that neural crest cells are collectively attracted toward the chemokine Sdf1. While not involved in initially polarizing cells, Sdf1 directionally stabilizes cell protrusions promoted by cell contact. At this cell contact, N-cadherin inhibits protrusion and Rac1 activity and in turn promotes protrusions and activation of Rac1 at the free edge. These results show a role for N-cadherin during contact inhibition of locomotion, and they reveal a mechanism of chemoattraction likely to function during both embryogenesis and cancer metastasis, whereby attractants such as Sdf1 amplify and stabilize contact-dependent cell polarity, resulting in directional collective migration.

## Graphical abstract

**Graphical Abstract:**

**Figure:**
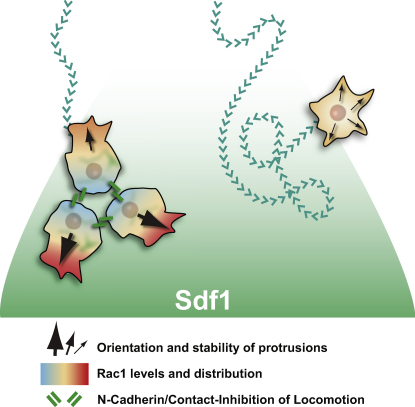

**Highlights:**

► Neural crest (NC) cells undergo collective chemotaxis toward Sdf1 ► N-cadherin-dependent contacts are required for NC cell chemotaxis toward Sdf1 ► N-cadherin regulates contact inhibition of locomotion by controlling small GTPases ► Sdf1 reinforces cell polarity induced by N-cadherin/contact inhibition

## Introduction

Although individual cell migration often involves an epithelial-to-mesenchymal transition (EMT) during which cell-cell adhesion is thought to be down-regulated (Thiery and Sleeman, 2006), many cell types undergo migration as coherent groups both during embryonic development and in metastatic cancers. Prior to and during collective cell migration, recent data suggest that cell-cell adhesion molecules may establish cell polarity (Desai et al., 2009; Dupin et al., 2009; Rieger et al., 2009). Such collective cell migration has been widely proposed as a common mechanism of invasion of numerous tumors (Bidard et al., 2008; Christiansen and Rajasekaran, 2006; Friedl and Wolf, 2003) and was recently observed in vivo in breast cancer cells (Giampieri et al., 2009). It also reflects cell behaviors during a number of developmental events ranging from lateral line migration in zebrafish to border cell migration in *Drosophila* (Friedl and Gilmour, 2009; Rorth, 2009). Cell clusters are more than a juxtaposition of individual cells. Contact inhibition of locomotion (CIL) within the group helps establish polarity at the leading edge (Carmona-Fontaine et al., 2008). Thus, cell-cell contacts appear to play an active role in cell migration. However, the molecular mechanisms underlying this cell behavior and particularly those conferring directionality during collective migration remain unclear.

External factors such as chemorepellents and chemoattractants have been proposed to confer directionality onto migratory cell populations. For trunk neural crest (NC) cells, both ephrins and semaphorins appear to restrict NC cells to the rostral half of each somite (Kuriyama and Mayor, 2008), resulting in a segmental pattern of migration. In contrast, less is known about attractive signals for the neural crest. One factor that has been proposed to attract NC cells is the chemokine Sdf1 (Belmadani et al., 2005; Olesnicky Killian et al., 2009). However little is known about how this, or other attractive signals, can be integrated by a migratory group.

During chemotaxis, cells must couple the sensing of extracellular chemoattractant with intracellular reorganization to allow directional migration (Andrew and Insall, 2007; Arrieumerlou and Meyer, 2005; Brahmbhatt and Klemke, 2003). It remains controversial whether chemoattractants induce localized formation of cell protrusions or simply provide a bias to the lifetime of random protrusions (Andrew and Insall, 2007; Iglesias and Devreotes, 2008). Despite their critical implications in cell migration, little is known about the putative interplay between cell interactions occurring during collective migration and chemotaxis.

Here, we study the mechanism of chemotaxis and the driving force of directional collective migration using *Xenopus* NC cells as a model. In *Xenopus*, cephalic NC cells start their migration as a cohesive cell population before progressively dissociating as individual cells (DeSimone et al., 2005; Sadaghiani and Thiebaud, 1987). We show that groups of NC cells are attracted from a distance to a source of Sdf1 which amplifies and stabilizes protrusions that are established at the leading edge by an N-cadherin/CIL-dependent mechanism. This combined mechanism breaks the symmetry of the group and leads to directional migration in response to Sdf1 gradient. Importantly, cell contacts dependent polarity is required for efficient chemotaxis as cell dissociation or inhibition of N-cadherin impairs chemotaxis. Altogether these results indicate that even if Sdf1 signaling is received at the single cell level it is only translated in directional information when cells have N-cadherin-dependent interactions. We propose to name “collective chemotaxis” to this collective interpretation of a chemotactic gradient.

## Results

### Sdf1-Cxcr4 Axis Is Required for NC Migration In Vivo

**Figure 1:**
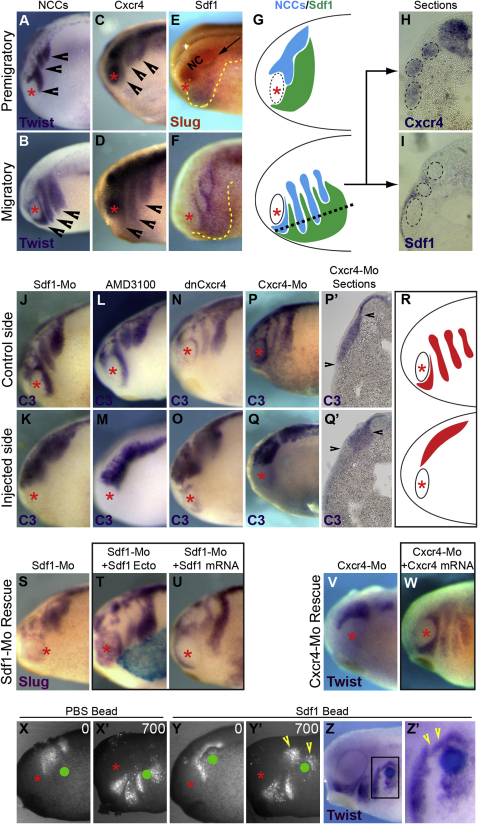
Sdf1-Cxcr4 Axis Is Required for NC Migration In Vivo (A–H) Premigratory ([A], Twist) and migratory ([B], Twist) NC cells (arrowheads) express Cxcr4 (C and D) and are surrounded by Sdf1-expressing ectoderm ([E and F], arrow, yellow dotted lines). (G) Summary of NC cells and Sdf1 distribution at premigratory and migratory stage. (H and I) Sections showing Cxcr4 expression in NC cells (H) and Sdf1 in the adjacent ectoderm (I); NC cells streams are delimited by dashed circles. (J–R) Embryos injected with Sdf1-Morpholino ([J and K], n = 132), treated with Cxcr4 inhibitor AMD3100 ([L and M], n = 128), injected with dominant-negative Cxcr4 (N and O, n = 77) or Cxcr4-Mo ([P and Q], n = 119) show clear inhibition of neural crest migration on the experimental side. (P′–Q′) Sections of Cxcr4-Mo-injected embryo. Arrowheads indicate border of the neuroepithelium and the front of NC cells migration. (R) Summary of phenotype after inhibition of Sdf1-Cxcr4 axis. (S–U) Rescue of Sdf1 inhibition by graft of Sdf1-expressing ectoderm ([T], n = 20) or coinjection of Sdf1-Mo and Sdf1 mRNA ([U], n = 68). (V and W) Rescue of Cxcr4-Mo by coinjection of Cxcr4-Mo and Cxcr4 mRNA (n = 14). (X–Z′) NC cells labeled with nRFP were grafted along side PBS ([X and X′], n = 4) or Sdf1 beads ([Y and Y′], n = 4). (X and Y) Frames of time-lapse movies showing in vivo NC migration. Green dot, grafted bead. Note that normal NC migration (X′) is partially affected by Sdf1 beads (Y′), with cell accumulating around the bead (arrowhead). (Z and Z′) Embryos analyzed by *Twist* in situ hybridization after graft of Sdf1 beads show ectopic NC cells located in between the streams (Z and Z′, arrowheads, n = 2).

As previous studies involved Sdf1 signaling in regulating NC cells migration in zebrafish and mouse embryos (Belmadani et al., 2005; Olesnicky Killian et al., 2009), we first checked that Cxcr4 and Sdf1 were respectively expressed in *Xenopus* NC cells and their surrounding tissues during migration. Comparison of NC markers at the premigratory and migratory stages (Figures 1A and 1B) with that of Cxcr4 (Figures 1C, 1D, and 1H) confirms that *Xenopus* NC cells are expressing Cxcr4 prior to and during migration. In addition, Sdf1 is expressed in the ectoderm facing NC cells before the onset of migration (Figures 1E, 1G, and 1I) and at the front and in between the migrating streams as migration proceeds (Figures 1F, 1G, and 1I). To confirm that Sdf1-Cxcr4 axis is required for NC migration in vivo, we performed a series of loss-of-function using Sdf1-Morpholino (Figures 1J and 1K), AMD3100, a specific chemical inhibitor for Cxcr4 (Figures 1L and 1M), a dominant negative for Cxcr4 (dnCxcr4, Figures 1N and 1O), and Cxcr4-Morpholino (Figures 1P–1Q′). All these treatments induced a strong inhibition of NC migration with injected cells accumulating next to the neuroepithelium (Figures 1Q′ and 1R), while control cells were efficiently reaching ventral regions (Figures 1P′ and 1R). To further confirm the specificity of these treatments, we rescued the migration of Sdf1-Mo and Cxcr4-Mo-injected cells by respectively grafting a piece of ectoderm overexpressing Sdf1 (Figures 1S and 1T) or coinjecting Sdf1 mRNA in the ectoderm (Figure 1U) or Cxcr4 mRNA (Figures 1V and 1W) alongside the Morpholinos. Finally, grafts of beads soaked in Sdf1 induce ectopic migration of NC cells in between the streams (Figures 1Z and 1Z′, arrowheads) or cause NC cells to stop their migration around the bead instead of migrating further ventrally (Figures 1Y and 1Y′, arrowheads), while PBS beads have no effect on the pattern of NC migration (Figures 1X and 1X′). Altogether these data indicate that Sdf1-Cxcr4 axis is required for directional migration in vivo of *Xenopus* neural crest, making these cells a good model to further investigate the role of Sdf1 in regulating directional migration.

### Cell Interactions Are Essential for Chemotaxis toward Sdf1

**Figure 2:**
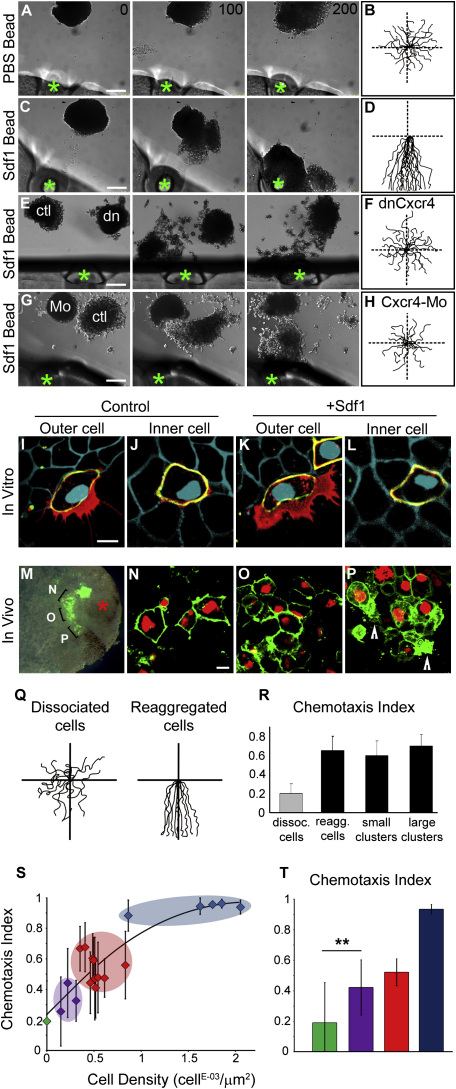
Cell Interactions Are Essential for NC Cell Chemotaxis toward Sdf1 (A–H) In vitro attraction assay with control NC explant exposed to beads (green asterisk) soaked in PBS (A and B) or purified Sdf1 (C–H). (E and F) Control (ctl) and dominant-negative Cxcr4 (dn) explants compete over an Sdf1 bead (n_ctl_ = 25; n_dnCxcr4_ = 32). (G and H) Control (ctl) and Cxcr4 Morpholino-injected explants (Mo) compete over an Sdf1 bead (n_ctl_ = 12, n_Mo_ = 12). Tracks are shown at the right. (I–L) In vitro, cells were labeled with mbRFP (blue) and mosaic labeling of NC with mbGFP/nRFP. Optical sections of GFP mosaic labeled NC from the plane of the substrate (red, cell protrusions) and from 5 μm above (green, cell body) were overlaid with mbRFP image (blue, surrounding cells). Outer (I and K) and inner (J and L) cells showing no cryptic protrusions in between the cells and no influence of Sdf1 on the cluster organization. (M–P) In vivo, confocal images of migratory NC cells labeled with mbGFP and nRFP grafted in a control embryo. Not all cells are labeled. Early migrating cells located near the neuroepithelium show an epithelial-like organization with clear cell-cell boundaries (N). Cells in the middle of a stream show a more mesenchymal phenotype but have no clear protrusions in the cell contact region (O), while cells at the front of a migrating stream facing a free space have protrusions ([P], arrowheads). (Q and R) Tracks of dissociated and reaggregated cells (Q) and Chemotaxis index of cells dissociated, reaggregated, and small and large clusters (R). (S and T) Chemotaxis index of single cells (green, n = 25), single cells having transient contacts (purple, n = 21), single cells interacting with small clusters (red, n = 88), and large clusters (blue, n = 41). (T) Average chemotaxis index for each category analyzed in S (^∗∗^p < 0.01). Chemotaxis efficiency improves as cell density increases. Time in minutes. Scale bars in (A–H), 150 μm; (I–L), 10 μm. Error bars show standard deviation. See also Figure S1 and Movies S1–S4.

To determine if Sdf1 was able to act as a chemoattractant for NC cells, we designed two in vitro chemotaxis assays. In brief, heparin-acrylic beads are soaked in purified Sdf1 solution and either immobilized using high vacuum silicone grease or left free to move in proximity of the cells (See Experimental Procedures for details). Immobilized beads (green asterisk in Figures 2A, 2C, 2E, and 2G) soaked in Sdf1 attract NC cells, which display highly directional migration (Figures 2C and 2D; see Movies S1 and S2 available online), while NC expressing dnCxcr4 (Figures 2E and 2F), Cxcr4-Mo (Figures 2G and 2H), or exposed to PBS beads (Figures 2A and 2B) spread radially (Movie S1). In addition, in an assay in which beads can move freely after being pushed by the cells, Sdf1 beads are actively tracked by groups of NC cells which change their direction of migration to follow the movements of the beads, whereas PBS beads are ignored (Movie S3). Our results show that Sdf1 is a NC chemoattractant.

In our in vitro chemotaxis assay, NC cells appear to move toward Sdf1 in dense groups. To distinguish whether cells were migrating as an organized cluster versus as individuals in close proximity, we labeled cells in a mosaic fashion with a combination of membrane-GFP (mbGFP), nuclear-RFP (nRFP), and membrane-RFP (mbRFP) and examined cell protrusions by confocal microscopy. Interestingly, only outer cells at the border of the group had large protrusions at the free edge (Figure 2I), whereas inner cells had very small and transient protrusions, the size and duration of which is negligible compared with the protrusion at the free edge (Figure 2J) (size of protrusion for outer cells: 93 ± 8 μm^2^; inner cells: 5 ± 2 μm^2^). These are likely to be similar to cryptic protrusions described elsewhere (Farooqui and Fenteany, 2005; Vasilyev et al., 2009). Importantly, this organization of outer and inner cells was not affected by exposure to Sdf1 (Figures 2K and 2L). That only outer cell are polarized was confirmed by analyzing the dynamic of microtubules showing that only outer cells had the centrosome off-centered and microtubules growing preferentially toward the free-edge (data not shown). We also looked at NC cells migrating in vivo by confocal microscopy (Figures 2M–2P, not all cells are labeled) and confirmed that there are no protrusions between the cells when they are in close proximity (Figure 2N) even if the population gets progressively looser as migration proceeds (Figure 2O). However protrusions and high membrane activity can be observed when cells face a free space like at the front of the migrating stream (Figure 2P, arrowheads) confirming our in vitro observation.

We next tested whether organization as a group was required for Sdf1-dependent attraction by comparing the attraction of dissociated and reaggregated cells toward Sdf1. The dissociation/reaggregation cycle has no effect on cell migration or chemotaxis and reaggragated cells were attracted as efficiently as nondissociated control groups indicating that shuffling the cells does not impair the response of the group (Figures 2Q and 2R). However, a dramatic reduction in chemoattraction was observed in dissociated cells compared with groups (Figures 2Q and 2R). We then looked at the migration of isolated small clusters of NC cells to see if a critical size of the group was required. Importantly, small clusters of only two or three cells were responding as efficiently as reaggregated and nondissociated clusters of hundred of cells (Figure 2R). In addition, the organization of these small clusters is not fixed. Cells are not organized as small chains of front, middle, and rear cells that would allow them to sample a bigger portion of the gradient but exchange positions over time (Figure S1A). Moreover, cells show protrusions that are not properly oriented toward Sdf1 (Figure S1A), indicating that they do not chemotax more efficiently because of a better alignment along the gradient. The fact that reaggregated cells and small or large clusters showed similar chemotaxis abilities while single cells were not responding efficiently to Sdf1 suggested that cell interactions were important for chemotaxis. The question remains as if cells need to interact, even transiently, with other cells or to be part of group with stable cell contacts to chemotax toward Sdf1. To address this point, we analyzed chemotaxis to Sdf1 after cell dissociation in three different situations (Figures 2S and 2T; tracks in Figure S1B): at low cell density with isolated single cells having no contact with other cells (green), individual cells having only transient interactions (purple), and at high cell density with individual cells and small clusters interacting with each other (red). Interestingly, chemotaxis becomes more efficient as cell density increases (Figures 2S and 2T; Movie S4). More importantly, individual cells that have only transient contacts with other cells and that are never part of a cluster show a much better response than isolated cells indicating that only transient cell contacts are sufficient to restore the response to Sdf1. The lower chemotaxis of these individual colliding cells compared with front cells from clusters with permanent contacts is likely due to the fact that a transient contact is not as efficient in maintaining directionality as a more permanent cell interaction. Altogether these results indicate that single NC cells do sense Sdf1 but can only efficiently interpret the gradient if they have interactions with other cells. In addition, even if cell coordination and chemotaxis are more efficient in groups, no specific size seems to be required as small and large clusters show similar chemotaxis abilities.

### NC Cells Exhibit Collective Chemotaxis

**Figure 3:**
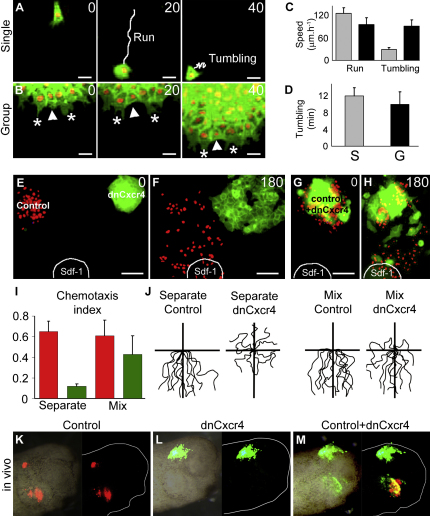
Cell Cooperation Accounts for Efficient Collective Chemotaxis (A–D). Run and tumbling of NC cells. Single cell (A) and cells in a cluster (B) exposed to a source of Sdf1 (at the bottom) show alternation of run and tumbling. Asterisks indicate cell protrusions; white arrowhead marks a cell collapsing protrusion while moving forward. Time in minutes. Scale bars, 20 μm. (C and D) Migration speed during run and tumbling ([C], gray bars, single cells; black bars, groups) and tumbling duration (D). (E–M) Rescue of nonresponsive Sdf1 cells by wild-type cells. (E and F) Control NC cells (red nucleus, n = 6) or dnCxcr4 (green membarne; n = 6) were separately exposed to Sdf1. (G and H) Mix of control (red nucleus) and dnCxcr4 (green membrane) exposed to Sdf1 (n = 20). (I) Chemotaxis index of separated or mixed control (red bars) and dnCxcr4 (green bars) cells. (J) Tracks of NC cells shown in (E) –(H) as indicated. Time in minutes. Scale bars, 150 μm. (K–M) NC migration in vivo. Host NC cells were removed and replaced by control (K) or dnCxcr4 (L) NC cells, or both (M). Error bars show standard deviation. See also Figure S2 and Movies S5 and S6.

Our observations show that NC cells respond to Sdf1 more efficiently as a collective than as individual cells. Migration of individual cells has been characterized by an alternation between two phases: run and tumble (Polin et al., 2009; Potdar et al., 2009). The run corresponds to a phase of directional migration, while the tumble is described as a reorientation phase characterized by collapse of protrusions and a series of short, randomly oriented movements, with no net migration. This behavior can be observed in dissociated cells or cells in groups by looking at the collapse of cell protrusions (Figures 3A–3D; Movie S5). While single cells almost completely stop during tumbling due to the collapse of protrusions, tumbling cells in a group are pulled by neighboring cells, retaining forward movement at the same speed (Figure 3C). Tumbling cells, since they collapse protrusions, can be considered as nonmotile cells for the period of the tumbling. Interestingly, dividing cells, which are not motile, keep moving forward at the same speed that nondividing cells pulled by the rest of the group (Figure S2A). Therefore, it is likely that this cooperation between cells inside a group accounts for the even flow of NC clusters toward Sdf1.

The observation of tumbling and dividing cells moving forward suggested that not all cells need to respond to Sdf1 for the group to undergo directional migration. To specifically address this point, we ran chemotaxis assays with control cells and cells expressing a dominant-negative of Cxcr4 (dnCxcr4), separately or mixed together. When mixed, dnCxcr4 cells interacting with control cells are able to migrate toward Sdf1 (Figures 3E–3J; Movie S6). Similar results were obtained in vivo (Figures 3K–3M). These results suggest a non-cell autonomous behavior such as not all cells in a group need to respond to Sdf1, similar to lateral line migration in zebrafish (Haas and Gilmour, 2006). However, even if not all the cells need to respond to the chemoattractant, one could hypothesize that nonresponding cells may help conferring a clear front-back polarity at the group level. We therefore monitored cells at the front and at the back of an attracted explant. All cells were producing protrusions toward the free space regardless of the orientation of the gradient. In fact, cells at the back produce protrusions oriented opposite to the gradient (Figures S2B–S2H). In addition, when considering large groups of NC cells exposed to Sdf1, the front of an explant evenly progresses forward while cells at the back show chaotic movement with phases of net movement backward, forward, or stagnation. This shows that cells at the back do not behave according to Sdf1 gradient and argues against the possibility that these back cells may contribute to the global polarity of the explant. Altogether these results indicate that NC cells are undergoing collective migration during which responding cells help nonresponding cells to move forward. This is likely to explain the more efficient chemotaxis of cell clusters.

### Sdf1 Amplifies Contact-Dependent Cell Polarity

**Figure 4:**
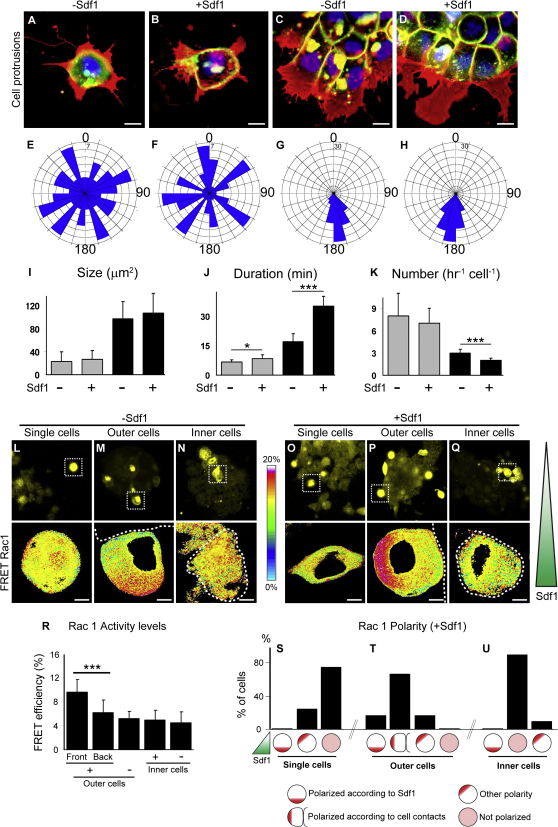
Sdf1 Stabilizes Cell Polarity Induced by Cell Interactions (A–D) Two-plane confocal image to show cell protrusions (red) and cell shape (green) in single cells (A and B) and groups (C and D), with (+) or without (−) Sdf, as indicated. (E–H) Orientation of cell protrusions analyzed from time-lapse movies in single cells (E and F) and groups (G and H), with (F and H) or without (E and G) Sdf1. (I–K) Size (I), duration (J), and numbers (K) of protrusions are shown for each condition (n = 50 per condition). Gray bar, single cells; black bar, group of cells. ^∗^p < 0.05; ^∗∗∗^p < 0.005. (L–U) FRET analysis of Rac1 activity in single, outer, and inner cells without (L–N) and with (O–Q) Sdf1 shows that Rac1 activity distribution is depending on cell contacts. (R) Levels of Rac1 activity in outer cells at the front (n = 26) or at the back (n = 25) of an explant with (+) or without (−, n = 20) Sdf1 and inner cells with (+, n = 6) or without (−, n = 6) Sdf1. ^∗∗∗^p < 0.005. (S–U) Summary of Rac1 activity distribution in single (S), outer (T), and inner (U) cells exposed to Sdf1. Circles under each bar represent different types of Rac1 polarities, which were quantified. Error bars show standard deviation. See also Figure S3.

To distinguish the respective roles of chemoattraction and cell-cell contact during directional migration, we analyzed the effect of Sdf1 exposure on single cells versus groups. Individual cells produce a large number (Figure 4K) of small (Figure 4I), unstable (Figure 4J) protrusions in all directions (Figures 4A and 4E), while cells in a group have a few (Figure 4K), large (Figure 4I), well-oriented (Figures 4C and 4G), and stable (Figure 4J) protrusions. Strikingly, the size and orientation of protrusions appear to be independent of Sdf1 exposure (Figures 4B, 4F, 4D, and 4H–4K; see Movies S2 and S4 for typical single cells and cell groups behaviors with or without Sdf1), indicating that the chemoattractant Sdf1 does not promote formation of oriented cell protrusions. Instead, the random orientation of cell protrusions in isolated cells suggests an intrinsic mechanism for the production of cell protrusions. Cell clustering is both necessary and sufficient to induce a strong front-back cell polarity, evidenced by the formation of large, well-oriented, stable protrusions at the front. Interestingly, Sdf1 slightly stabilizes protrusions in single cells (Figure 4J, gray bars) and strongly stabilize them in groups (Figure 4J, black bars). These data, alongside our previous observation that cells at the back produce protrusions against the gradient, indicate that the stability of the protrusions is controlled primarily by cell-cell contacts and partially by Sdf1, while the size and the orientation of the protrusions only depend upon cell clustering. However, it remains possible that Sdf1 may not directly stabilize protrusions. In fact, when cells are exposed to Sdf1 they move toward the same point, thus reducing the probability of collisions while in control explants random movements may lead to a higher rate of cell collisions and protrusions collapse. If true, such differences in terms of cell coordination could explain the difference in terms of cell protrusions. To address this point, we first compared protrusions stability at the border of control explants that were spreading randomly in all direction and in control explants that were spontaneously moving in one direction. Importantly, we found no difference in protrusions stability between the two conditions (Figures S3A–S3G), indicating that cell alignment is not sufficient to increase protrusions stability. We then compared protrusions stability in a mosaic of control and dnCxcr4 cells exposed to Sdf1 (Figures S4H and S4I). Protrusions in control cells had a high stability while protrusions in dnCxcr4 cells had a low stability similar to that of control cells in absence of Sdf1. These data further reinforce the idea that high protrusion stability observed at the front of an explant migrating toward Sdf1 is due to a direct effect of the chemokine on cell protrusions and is not a side effect of cell coordination occurring during directional movement.

Some of the main regulators of protrusions formation and stability are the small Rho GTPases, Rac1 and Cdc42 (Ridley et al., 2003). We have recently showed that Rac1 plays a major role on NC migration, while we found no evidence for Cdc42 (Carmona-Fontaine et al., 2008; Matthews et al., 2008). Therefore, we examined the respective influence of Sdf1 and cell contacts on Rac1 activity and localization using FRET analysis. Importantly, outer cells in control (Figure 4M) and Sdf1 conditions (Figure 4P) have a clearly polarized Rac1 activity distribution with low levels at the regions of cell-cell contacts and high levels at the free edge. In contrast single cells, with no contacts with other cells, or inner cells, that are completely surrounded, show no obvious polarity (Figures 4L, 4N, 4O, and 4Q). Seventy-five percent of single and 90% of inner cells show no Rac1 polarity (Figures 4S and 4U), while 67% of outer cells are polarized according to the cell contact (Figure 4T) and less than 20% according to Sdf1. These results show that the distribution of Rac1 activity inside the cells is depending on cell-cell contacts and not on Sdf1. Despite its lack of effect on Rac1 distribution, Sdf1 amplifies the polarity of cells at the front of an explant by further increasing Rac1 activity at the free edge (Figure 4R, compare Figures 4M and 4P) but has no influence on Rac1 activity in inner cells (Figure 4R). These data suggest that Sdf1 breaks the radial symmetry of the group and polarizes the explant by stabilizing protrusions at the front.

### N-Cadherin Mediates Cell Interactions during NC Cell Migration

**Figure 5:**
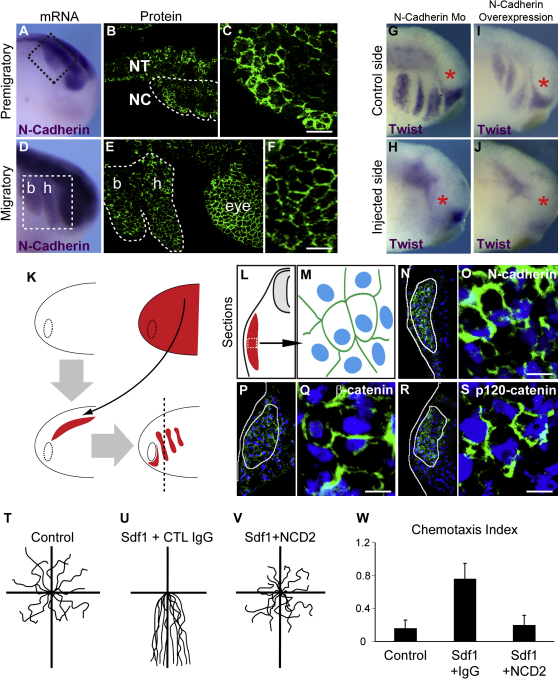
N-Cadherin-Dependent Contacts Are Required for Collective Chemotaxis (A–F) N-cadherin expression in premigratory (A–C) and migratory (D–F) NC cells analyzed by whole mount in situ hybridization (A and D) and immunostaining (B, C, E, and F); NC cells streams are delimited by dotted lines. (G and H) N-cadherin loss-of-function using an antisense Morpholino (n = 87). (I and J) Full-length N-cadherin overexpression (n = 40). (K–S) NC cells labeled with rhodamine-dextran (RD) were grafted into unlabeled embryos and NC migration was monitored looking at the RD fluorescence. Immunostaining on sections for N-cadherin (N and O), β-catenin (P and Q), and p120-catenin (R and S) are shown in low and high magnification. Blue, DAPI staining. Scale bar, 20 μm. (T–V) Tracks of control cells ([J], n = 16) and NC pretreated with a control IgG ([K], n = 22) or with N-cadherin blocking antibody NCD2 ([L], n = 27) and exposed to Sdf1 showing that N-cadherin inhibition strongly blocks chemoattraction toward Sdf1. Chemotaxis index for each condition is shown in (W). b, branchial; h, hyoid. Error bars show standard deviation. See also Movies S7 and S8.

Previous observations in other species (Bronner-Fraser et al., 1992; Monier-Gavelle and Duband, 1995; Theveneau et al., 2007; Xu et al., 2001) have shown that some migratory neural crest populations express N-cadherin. Our findings show that premigratory and migratory *Xenopus* NC cells express N-cadherin at both mRNA and protein levels in vivo (Figures 5A–5F), making it a good candidate for mediating NC cell interactions. To test whether N-cadherin plays a functional role during migration, we inhibited it by using an antisense Morpholino (Nandadasa et al., 2009). The results show dramatic effects on cell migration (Figures 5G and 5H). In the reciprocal experiment, overexpression of full-length N-cadherin blocked NC migration (Figures 5I–5J). These data show that the levels of N-cadherin must be correctly regulated in order to allow for proper NC migration. To confirm that N-cadherin was involved in functional cell junctions in migratory NC, we grafted neural crest labeled with rhodamine-dextran into unlabeled host in vivo (Figures 5K–5M) and monitored N-cadherin (Figures 5N and 5O), β-catenin (Figures 5P and 5Q), and p120-catenin (Figures 5R and 5S) localizations (Movie S7). All these factors were observed at regions of cell contact between labeled migrating neural crest cells supporting the idea that N-cadherin is involved in functional cell-cell contacts during migration.

We next performed the fixed bead chemotaxis assay in the presence of N-cadherin-blocking antibody (NCD2, Takeichi, 1988) or a control IgG. Interestingly, we found that NC cells that were preincubated with NCD2 showed a dramatic loss of attraction toward Sdf1 (Figures 5V and 5W) compared with controls that underwent collective migration toward Sdf1 (Figures 5U and 5W) and were similar to cells spreading randomly in absence of Sdf1 (Figures 5T and 5W; Movie S8).

**Figure 6:**
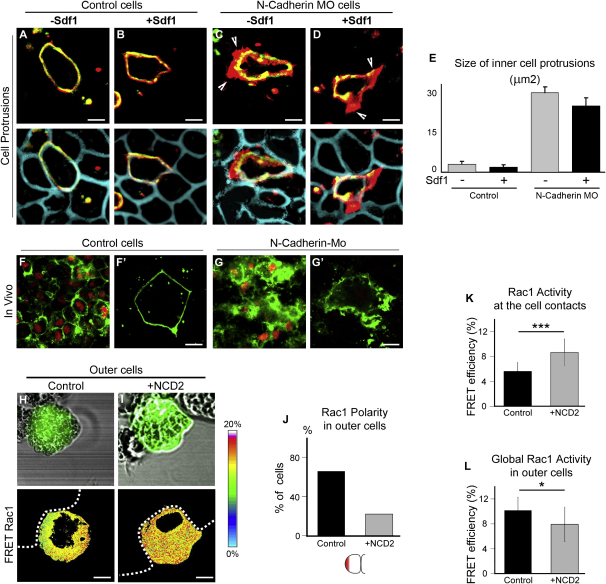
N-Cadherin-Dependent Cell Interactions Prevent Formation of Cell Protrusions through Local Inhibition of Rac1 at Cell Contacts (A–D) Embryos were injected with mbRFP (blue) at the 2 cell stage and with N-cadherin MO/mbGFP at the 32 cell stage to generate a mosaic expression of the MO. Two-plane confocal image to show cell protrusions (red) and cell shape (green) in control cells (A and B) and N-cadherin MO cells (C and D), with (+) or without (−) Sdf1, as indicated. N-cadherin loss-of-function induces formation of ectopic cell protrusions overlapping with neighboring cells ([C and D], arrowheads) regardless of Sdf1. (E) Size of inner cell protrusions (n = 10). (F–G′) In vivo, confocal images of migrating NC cells labeled with mbGFP and nRFP grafted into a control embryo. Not all the cells are labeled. Region shown equivalent to Figure 2O (middle of NC stream). Control cells (F and F′) have clear cell-cell boundaries while N-cadherin-Mo-injected cells (G and G′) have high membrane activity and show overlapping protrusions. Labeled cells surrounded by nonlabeled cells are presented in high magnification in F′ and G′. Scale bars, 15 μm. (H–J) FRET analysis of Rac1 activity distribution in control outer cells or outer cells treated with NCD2 antibody as indicated (n = 27). Scale bars, 10 μm. (K) Rac1 activity at the cell contacts region (n = 18). (L) Global Rac1 activity in outer cells (n = 29) ^∗^p < 0.05, ^∗∗∗^p < 0.005. Error bars show standard deviation. See also Figure S4 and Movie S9.

Because efficient chemotaxis requires contact-mediated inhibition of cell protrusions at the cell contact region, we hypothesized that N-cadherin may be involved in this process. To test this, we first injected NC cells with N-cadherin MO and looked at cells protrusions during cell migration. Morpholino-injected cells were highly motile, dispersed quicker than controls, and produced numerous protrusions (Movie S9). Importantly, morphant cells produce protrusions on top of each other, and wide overlapping between the cells is observed (Figure S4). This indicates that N-cadherin inhibition directly affects the ability of the NC cells to sense each other. In contrast, control cells had a more steady behavior, low migratory activity, and no apparent cell protrusions between them (Figure S4; Movie S9). To substantiate this, we created cell mosaics by injecting mbGFP and mbRFP at different stages and looked at morphant and control cells surrounded by other control cells. As described above (Figure 2), control inner cells exhibit very small cryptic protrusions (Figures 6A and 6B). However, N-cadherin MO-injected inner cells showed clear cell protrusions regardless of Sdf1, indicating that these cells had lost the ability to inhibit protrusions by cell contact (Figures 6C–6E). We confirmed this observation in vivo by confocal microscopy (Figures 6F–6G′). NC cells injected with N-cadherin Morpholino are disorganized and have diffused cell-cell boundaries with overlapping regions (Figures 6G and 6G′), whereas controls cells show a clear cell contact region (Figures 6F and 6F′). Furthermore, we found that NCD2 treatment dramatically reduced Rac1 polarity in the outer cells (Figures 6H–6J) and increased Rac1 levels at the region of cell contacts (Figure 6K). Interestingly, the global levels of Rac1, including the front, were reduced (Figure 6L), suggesting some kind of positive feedback loop from back to front related to the polarized distribution of Rac1.

Altogether these results indicate that N-cadherin-dependent cell contacts polarize the cells by inhibiting Rac1 at the cell contact and increasing Rac1 activity at the free edge; this polarized distribution of Rac1 activity appears to be essential for the cells to respond to a chemoattractant.

### N-Cadherin-Dependent Cell Contacts Are Required for Contact Inhibition of Locomotion

**Figure 7:**
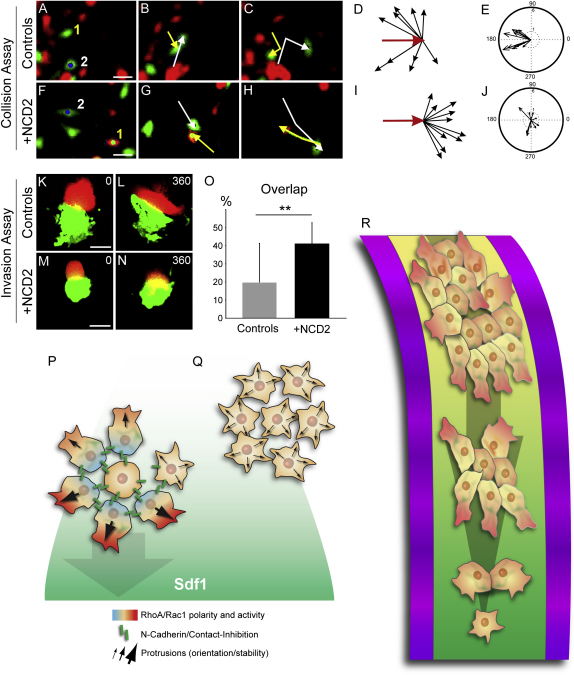
N-Cadherin Is Required for CIL (A–J) Collision assays between control (A–E) and NCD2-treated NC cells (F–J). Velocity (D–I) and acceleration (I–J) vectors for control (D and E) and NCD2-treated cells (I–J). Note the clear change in direction of migration upon collision in control cells (p < 0.005, n = 10) is lost in NCD2-treated cells (n = 10). (K–O) Invasion assays between control NC cells explants ([K and L], n = 36) and NCD2-treated explants (M and N, n = 47). Control explants do not invade each other (L and O), whereas N-cadherin inhibition allows NC cells to invade each other (O). (P–R) Model for *Xenopus* NC cells collective chemotaxis. The color gradient in the cytoplasm represents the levels and distribution of Rac1 (red) and RhoA (blue, after Carmona-Fontaine et al., 2008; Matthews et al., 2008) activities. The different thicknesses and directions of the arrows indicate the relative stabilities and orientation of protrusions, respectively. N-cadherin is represented as a green bar. Nuclei are shown as gray circles and the external gradient of Sdf1 as shades of green. (P) NC cells clusters exhibit radial symmetry where all outer cells are polarized with protrusions toward the free edge and inner cells are not polarized. When exposed to a gradient of Sdf1, protrusions at the front are further stabilized and the initial radial organization is broken leading to directional migration. (Q) If cell interactions are prevented (N-cadherin inhibition, cell dissociation), Rac1 distribution no longer matches cell-cell interactions and global levels are lowered thus inducing protrusions instability, loss of coordination among the cells, and the loss of directional migration. (R) Representation of the NC cells migration in vivo where NC cells are maintained on migratory routes by inhibitory cues (shades of purple) and attracted ventrally by chemotaxis to Sdf1. Protrusions can be seen at the border of the group and in between the cells only when gaps are generated. The NC cells population gets looser as migration proceeds ventrally and progressively breaks away as single cells. Error bars show standard deviation. See also Movie S10.

We recently described that the formation of cell protrusions in between NC cells is prevented by CIL mediated by the Wnt/PCP pathway (Carmona-Fontaine et al., 2008). When CIL is abolished, a dramatic increase of the size of the cryptic protrusions was observed. As we obtained a similar effect after N-cadherin inhibition, we decided to test whether N-cadherin was involved in CIL. Two main methods were originally used to analyze CIL: single cells collisions assays and explants invasion assays (Abercrombie and Heaysman, 1953; Carmona-Fontaine et al., 2008). We used both to address N-cadherin requirement in CIL. As expected, collisions between control NC cells lead to a dramatic change of direction (Figures 7A–7E; Movie S10) while after inhibition of N-cadherin cells ignore each other showing no change of direction after contact (Figures 7F–7J; Movie S10). In addition, control NC explants cannot efficiently invade each other (Figures 7K, 7L, and 7O, gray bar; Movie S10). However, when one or both explants were treated with NCD2, NC cells groups were able to invade each other and widely overlapped (Figures 7M, 7N, and 7O; black bar, Movie S10). These data confirm that NC cells need functional N-cadherin to exhibit CIL.

In a normal context, NC cells experience the influence of CIL and external cues at the same time. We therefore decided to analyze the interplay between CIL and Sdf1 by analyzing collisions between controls cells in presence or absence of Sdf1 gradient. No differences were noted in between the two conditions (Figure S4). This suggests that the presence of a chemoattractant does not reduce the cell polarization induced by CIL, thus supporting the notion that cell polarity is mainly determined by cell contacts rather than by a chemoattractant. Furthermore, as all our treatments affecting cell contacts (dissociation, N-cadherin-Mo or NCD2 blocking antibody) also affect the ability of the cell to chemotax, our data emphasize the idea that collective chemotaxis is CIL dependent.

## Discussion

We have shown here that cell clusters exhibit radial polarity with large stable protrusions in the polarized outer cells and high level of Rac1 at the free edge and nonpolarized inner cells. This radial symmetry is broken upon addition of a chemoattractant that further stabilizes protrusions and increases Rac1 activity at the front, leading to directional migration of the cluster toward the source of the chemoattractant (Figure 7P). This cell polarity is N-cadherin/CIL dependent and essential for efficient chemotaxis (Figures 7P and 7Q). Inhibition of cell interactions leads to a loss of CIL resulting in a loose arrangement of cells with no difference between inner or outer cells, no stable cell polarity, and poor chemotaxis (Figure 7Q). These data, alongside in vivo description of *Xenopus* NC cells migration, suggest that these cells migrate as a cohesive cluster progressively breaking away as single cells, similar to the original description of cluster migration done by Trinkaus (Trinkaus, 1988). The effect of cell dissociation on chemotaxis in vivo may be counterbalanced by inhibitory cues at the border of the migration route in order to maintain the cells in close proximity (Figure 7R) or by a hypothetical attraction between the cells.

One of the big issues with collective movement is how the driving force is generated. While in small clusters like the *Drosophila* border cells it is possible that a couple of front cells may pull the rest of the group, it is unlikely that a few front cells would be sufficient to achieve the same effect in large populations like the NC. In fact, studies on cell sheet migration have shown that the main driving force arises from cells inside the group while leading cells are mainly giving direction (Trepat et al., 2009). However, despite the fact that we demonstrated the requirement of cell interactions, NC cells remain a mesenchymal population. Relative positions of a given cell and its direct neighbors are not fixed. Cells do exchange positions and gaps are constantly appearing in between the cells leading some inner cells to form protrusions and behaving as front cells for a while before colliding with the cells in front or next to them. These observations strongly indicate that the NC cells population should be seen as a relatively cohesive population progressively breaking up as a collection of small clusters of variable cell composition that are constantly splitting, colliding, and reassembling (Figure 7R), rather than as a group with stable organization over time in which a wide group of inner cells would have to be pulled by a few front cells. Consequently, we think that NC migration cannot be directly compared with epithelial movements during wound healing or lateral line migration in terms of physical motion of the group.

Another aspect of collective migration that studies on lateral line have highlighted is the possibility that inner cells could act as a sink by trapping Sdf1 using Cxcr7 and therefore helping to shape the gradient itself (Dambly-Chaudiere et al., 2007; Valentin et al., 2007). In this system, Sdf1 expression in the surrounding tissues is homogenous (David et al., 2002), making necessary an additional system like the sink model to shape a gradient along which the cells can move. On the contrary, in *Xenopus*, Sdf1 expression is progressively shifting ventrally and is constantly ahead of the NC cells position along the dorso-ventral axis. In addition, isolated small clusters, in which there are no inner cells, as they are all exposed to a free space and produce protrusions, migrate as efficiently as big groups. Moreover, transient contacts between single cells are sufficient to partially restore chemoattraction. All these observations indicate that a sink system similar to that described for the lateral line is unlikely to be required for NC cell migration. Although our results demonstrate a crucial role for cell interactions during NC directional migration, we can not exclude that other mechanisms, such as inner cell acting as sink for chemoattractant signals or a global detection of chemoattractants by the whole cluster, could also cooperate with CIL in vivo.

Different alternatives about how chemoattractant are generating directional migration have been proposed (Andrew and Insall, 2007; Iglesias and Devreotes, 2008). Some argue that chemokines induce the formation of cell protrusions and use the protrusions as a physical markers of responding cells (Haas and Gilmour, 2006), while others suggested that stabilization of cell protrusions formed independently of the chemotactic signaling could be sufficient to generate directional movement (Andrew and Insall, 2007). A recent study clearly showed that the increase of protrusion stability correlates with an increase in cell persistence (Harms et al., 2005), reinforcing the possibility that stabilizing pre-existing protrusions can lead to directional migration. Our data in NC cells support the notion that chemoattractants stabilize protrusions at the front of a cell cluster, creating an asymmetry and leading to directional migration of the cell group.

Our results on cell-contact-dependent polarity are consistent with our recent findings showing that activation of RhoA at regions of NC cell contact is essential for migration during CIL (Carmona-Fontaine et al., 2008), in which cell protrusions are inhibited after cell-cell contact (Abercrombie and Heaysman, 1953). Here we further show that cell contacts, CIL dependent and mediated by N-cadherin, are essential for NC chemotaxis and that the polarization of the small GTPases by cell contact is important for optimal response to a chemoattractant. Besides, we have shown here that N-cadherin is required for CIL at the cell contacts and that N-cadherin inhibition leads to an increase of Rac1 activity at the juxtamembrane domain probably due to a lack of RhoA activation downstream of the Wnt/PCP pathway. The precise mechanism of interaction between N-cadherin and Wnt/PCP during CIL remains to be investigated.

Our data also indicate that type I cadherin-mediated cell interactions are essential for proper collective migration of a highly mesenchymal and invasive cell population such as the neural crest. These data further support the idea proposed for cancer cells that transient epithelial-like cell interactions do not prevent mesenchymalization and migration (Yang and Weinberg, 2008). Interestingly, tip-like contacts were described during chick NC cells migration (Kulesa and Fraser, 2000; Teddy and Kulesa, 2004). We propose that such contacts could achieve the same effect on cell polarity that the pseudo epithelial-like interactions present in a migratory cohesive cell group. A possibility further supported by data showing that these cells exhibit CIL-like behaviors during migration (Kulesa and Fraser, 2000; Teddy and Kulesa, 2004). We show here that even invasive mesenchymal cells can benefit from cell-cell interactions, and it would be interesting to address the role of cell contacts during collective phase of cancer cell migration.

The results presented here may lead to the reinterpretation of recent studies. For example, inhibition of N-cadherin blocks directional migration of cerebellar granule neurons (Rieger et al., 2009) and LL (Kerstetter et al., 2004) consistent with our conclusion that cell contacts are required for directional migration and suggest that these phenotypes may be due to a loss in chemotactic response, as we have demonstrated for NC cells.

Finally our results, alongside the influence of CIL, give a more complete view of how large populations of cells can achieve directional migration by integrating cell interactions and external cues. They indicate that invasive cells need to interact not only with their local environment, but also with each other in order to migrate efficiently, and may give new angles to better understand and tackle invasive issues.

## Experimental Procedures

### Neural Crest Culture

In *Xenopus*, cranial NC cells are never part of the neuroepithelium and are located on the side of the neural plate. They are very cohesive at the beginning of migration and can be easily isolated as an explant (DeSimone et al., 2005; Sadaghiani and Thiebaud, 1987). They were dissected as described in (DeSimone et al., 2005). In brief, the pigmented epidermal layer is first removed then NC cells are gently taken out by microdissection. They stick to each other but barely attach to the neural plate or the mesoderm underneath. In addition, they can be easily distinguished from mesodermal cells that have a very strong white color while NC cells are transparent, slightly gray, and much smaller. This technique is easy to master and generally lead to pure NC cells culture. Explants contaminated with other cells types (e.g., mesoderm, ectoderm, or Rohon-Beard neurons) were ignored in this study. When needed, cell dissociation was performed by putting the NC explants in Ca^2+^/Mg^2+^-free medium for a few minutes before transferring them to normal culture medium.

### Chemotaxis Assays

A fixed beads assay was designed as an alternative for Boyden or Dunn chambers. It is suitable for high-resolution time-lapse microscopy and allows the use of a motorized stage for monitoring several explants at the same time. A free beads assay was designed as a variation of the pipette assay and is suitable to check if cells can change their direction of migration to track a moving source of chemoattractant. Both assays are easy to set up and can be adapted to any microscopic system at no extra cost.

### Preparation of Beads and Fibronectin-Coated Dishes

Heparin-acrylic beads (Sigma H5263, Adar Biotach 6024-1) were incubated for 1hr 30min in a 1 μg/ml Sdf1 solution in PBS and used to deliver Sdf1. Fibronectin (Fn, Sigma F2006) coating was done by incubating non-culture-treated plastic dishes at 37°C with a Fn solution at 10 μg/ml for 1 hr, washed with PBS, and incubated with PBS 0.1% BSA for a further 30 min.

### Protocol for the Fixed Beads Assay

The Fn coating was done first, the PBS/BSA solution was removed, and the dish was left to dry up for 2 min at room temperature. A line of silicone grease (VWR, 6366082B) was added inside the Fn region using a 20 ml syringe before adding the culture medium. A few incubated beads were placed in the dish outside the Fn region. Using an eyebrow knife, beads of a similar diameter (150–200 μm) were then moved inside the Fn region and positioned at the border of the grease before being pushed in using tweezers. The remaining beads were removed. The distance in between two consecutive beads was kept around 1 mm. The NC explants were dissected, placed in front of the beads in between 250 and 500 μm, and left to attach to the matrix for 30 min. The dish can then be filled up with an excess of culture medium and carefully closed by putting the lead back on (without letting air bubbles in). This is recommended if the dish has to be turned upside down to be used on an upright microscope. On the other hand, the dish can be left open and used with water immersion lenses or an inverted microscope. Both methods were successfully used. Competition assays between two kinds of cells can be easily run by putting two explants in front of the same bead. The response of the same kind of cells to two different conditions can be tested by placing different beads in the same dish (for instance PBS or Sdf1 beads) or by using multi-well dishes. For the latter, each well can be filled up by a different culture medium and sealed with a coverslip if necessary.

### Protocol for the Free Beads Chemotaxis Assay

The Fn coating was done first and explants were placed on the matrix and left to attach for 30 min. Incubated beads were added in the dish and kept outside the Fn region. A few of them were broken in pieces using tweezers. Pieces were selected and moved inside the Fn region using an eyebrow knife to be positioned where desired. The remaining beads were removed. Each piece is placed with the flat side in contact with the dish to help the beads to stay in place. This assay can be done without breaking the beads, but they therefore tend to move because of the Brownian movements in the liquid.

### Time Lapse, Tracking, and Cell Protrusion Analysis

Time lapse and tracking of migrating NC cells was performed as previously described (Carmona-Fontaine et al., 2008; Matthews et al., 2008). Tracking was made using ImageJ Manual Tracking plug-in. The tracks of each individual cells were represented in a graph with the origin at 0, 0, using MathLab or ImageJ Chemotaxis Tool plug-in. Cell protrusions were analyzed as described in Carmona-Fontaine et al. (2008) and Matthews et al. (2008). In brief, cell protrusions were defined by the positive difference in the area of a cell between two consecutive frames. Orientation of protrusion was determined by the vector between the centroid of the cell and the centroid of the protrusion using ImageJ. Visualization of cell protrusions was done by overlapping two plane focus from a confocal microscopy image (Carmona-Fontaine et al., 2008). Red was used for the substratum focus (protrusion) and green for the focus at the middle of the cell (cell body).

### FRET Analysis

Samples for analysis of FRET by acceptor photobleaching were imaged using a Zeiss LSM 510 META laser scanning confocal microscope and a 63× Plan Apochromat NA 1.4 Ph3 oil objective. The CFP and YFP channels were excited using the 440 nm diode laser and the 514 nm argon line, respectively. The two emission channels were split using a 545 nm dichroic mirror, which was followed by a 475–525 nm bandpass filter for CFP and a 530 nm longpass filter for YFP (Chroma). Pinholes were opened to give a depth of focus of 3 μm for each channel. Scanning was performed on a sequential line-by-line basis for each channel. The gain for each channel was set to approximately 75% of dynamic range (12-bit, 4096 gray levels) and offsets set such that backgrounds were zero. Time-lapse mode was used to collect one prebleach image for each channel followed by bleaching with 50 iterations of the 514 nm argon laser line at maximum power (to bleach YFP). A second postbleach image was then collected for each channel. Control nonbleached areas were acquired for all samples in the same field of view as bleached cells to confirm specificity of FRET detection. Pre- and postbleach CFP and YFP images were then imported into Mathematica 6 for processing. In brief, images were smoothed using a 3 × 3 box mean filter and background subtracted, and postbleach images were fade compensated. A FRET efficiency ratio map over the whole cell was calculated using the following formula: (CFP_postbleach_ − CFP_prebleach_)/CFP_postbleach_. Ratio values were then extracted from pixels falling inside the bleach region as well as an equally sized region outside of the bleach region and the mean ratio determined for each region and plotted on a histogram. The nonbleach ratio was then subtracted from the bleach region ratio to give a final value for the FRET efficiency ratio. Data from images were used only if YFP bleaching efficiency was greater than 70%.

### RNAs, DNAs, and Antisense Morpholinos Used for Microinjections

Antisense Morpholino were purchased from GeneTools: Cxcr4 (8ng, 5′-CAATGCCACCAGAAAACCCGTCCAT-3′), N-cadherin (8 ng) (Nandadasa et al., 2009), Sdf1-Mo (8ng, 5′-AGAGCTAGAGTCCTTATGTCCATGT-3′); mRNAs: dnCxcr4 (2 ng), Cxcr4 (500 ng) (Moepps et al., 2000), membrane-GFP (500 pg), membrane-RFP (500 pg), nuclear-RFP (500 pg), N-cadherin-GFP for localization (50 pg), full-length N-cadherin for overexpression (500 ng), p120 catenin-GFP (X. T. Zhao/A. B. Reynolds, 50 pg), Sdf1 (500 ng) (Braun et al., 2002); DNAs: Raichu-Rac1 (Itoh et al., 2002) FRET probe (75 pg). dnCxcr4 contains a single mutation replacing the tyrosine 194 by an alanine preventing Cxcr4 from being activated.

### mRNA Probes, Antibodies, and Proteins

*Xenopus* probes were as follows: C3 (McLin et al., 2008), Cxcr4 (Moepps et al., 2000), Sdf1 (Braun et al., 2002), N-cadherin (NIBB clone 403), Snail2 (Mayor et al., 1995), and Twist (Hopwood et al., 1989). Primary antibodies were as follows: β-catenin (AbCam, ab6302, 1:500), N-cadherin for immunostaining (DSHB, MNCD2 s, 1:2), N-cadherin for activity blocking purpose (Invitrogen, NCD2, 100 μg/ml); secondary antibodies were as follows: anti-rat IgG-FITC (Sigma, F6258, 1:200) and anti-rabbit IgG-FITC (Sigma F0382, 1:200). Human stromal cell-derived factor 1 was from Calbiochem (572300, 1 μg/ml).

### Histology, Immunostaining, In Situ Hybridization, FRET, and Antibodies

Cryosections and immunostaining on sections were performed as described in (Theveneau et al., 2007). *Xenopus* in situ hybridizations (ISHs) were performed as described in (Harland, 1991).

## Supplemental Information

Document S1. Four Figures

Movie S1. Chemotaxis toward Sdf1 Requires Cxcr4 Time-lapse movie of a fixed-bead chemotaxis assay with control NC cells confronted to PBS (top left) or Sdf1 bead (top right) and control NC cells competing with dnCxcr4 NC cells (bottom left) or Cxcr4-Mo NC cells (bottom right) in front of an Sdf1 bead. Only control NC cells exposed to Sdf1 are attracted. Without Sdf1 (top left) or when the response is inhibited by dnCxcr4 (bottom left) or Cxcr4-Mo (bottom right) no attraction is observed. One picture every 5 min. (Related to Figure 2.)

Movie S2. Comparison between Control NC Cells Spreading and NC Cells Attracted to Sdf1 Normal spreading of control NC cells (left) compared to control NC cells exposed to Sdf1 (right) in a fixed-bead chemotaxis assay. Cells were injected with nuclear-RFP and membrane GFP. Low magnification is shown to the right; rectangle indicates the higher magnification shown to the left. Cells exposed to Sdf1 undergo directional migration while control cells spread randomly from their original location. The net migration of the front when cells are exposed to Sdf1 is approximately 3× faster than when control cells spread randomly (110 μm/hr and 40 μm/hr, respectively). One picture every 5 min. (Related to Figure 2.)

Movie S3. Free-Bead Chemotaxis Assay with Sdf1 and PBS Beads Free-bead chemotaxis assay with control NC cells exposed to PBS and Sdf1 beads. Sdf1 beads are on the left-hand side, PBS beads on the right-hand side. Note that NC cells actively track Sdf1 beads. When a bead moves to a new location it induces a change in cells direction of migration. On the contrary, PBS beads are ignored. One picture every 5 min. (Related to Figure 2.)

Movie S4. Cell Contacts Are Required for Chemotaxis Comparison between isolated single cells without Sdf1 (first panel) or exposed to Sdf1 (second panel), single cells with transient contacts exposed to Sdf1 (third panel) and isolated small clusters exposed to Sdf1 (fourth panel). These movies emphasize the fact that cell interactions, even transient, restore efficient chemotaxis (third and fourth panels) while the lack of cell contacts leads to inefficient chemotaxis (second panel). (Related to Figure 2.)

Movie S5. Alternation of Run and Tumble in Isolated and Clustered Cells Run and tumbling behaviors in single cells and groups. Fixed-bead chemotaxis assay with Sdf1. Single cells alternate between two different behaviours: run and tumbling. Note that at the front of the group a continuous migration is observed even if cells also undergo tumbling and collapse protrusions at some points (white arrowhead). This movie also illustrates the lack of efficient chemotaxis of the single cells. Because of a poor directionality the single cell cross the field in more than twice the time as the group does. The group movie is played twice; the single cell movie is played once. One picture every 5 min. (Related to Figure 3.)

Movie S6. Cooperation between Responding and Nonresponding Cells Allows Nonresponding Cells to Migrate toward Sdf1 Control NC cells can rescue dnCxcr4 NC cells migration in a fixed-bead chemotaxis assay. When both cell types are separately exposed to Sdf1 (left), only the control NC cells (red nucleus) are attracted, while dnCxcr4 NC cells (green membrane) spread radially. Importantly, when mixed together with control NC cells, some dnCxcr4 NC cells are able to reach Sdf1 bead. dnCxcr4 cells in contact with control cells (groups 1 and 2) show a directional migration while dnCxcr4 losing contact with control cells (group 3) fail to maintain a directional movement. One picture every 5 min. (Related to Figure 3.)

Movie S7. N-Cadherin and p120 Localizations during NC Cells Migration Membrane localization of N-cadherin-GFP during NC cells migration in vitro (left). Membrane localization of p120-GFP during NC cells migration in vitro (right). One picture every 90 s. (Related to Figure 5.)

Movie S8. N-Cadherin Activity Is Required for Chemotaxis towards Sdf1 Fixed beads chemotaxis assay with control NC cells pre-treated with control IgG and anti N-Cadherin blocking antibody NCD2. NC cells treated with the control IgG show a normal response to Sdf1 and chemotax towards the source while NC cells treated with NCD2 show poor attraction. (Related to Figure 5.)

Movie S9. N-Cadherin Is Required for Contact Inhibition of Cell Protrusions at the Cell Contact Comparison between control NC cells and N-Cadherin MO injected cells. Control cells have clear cell-cell boundary while Morpholino-injected cells are more dispersed, overlap with their neighbours and can produce protrusions over pre-established contacts. Control cells: one picture per minute. Morphant cells: one picture every 2 min. The control movie is played twice faster to match the speed of the N-Cadherin MO movie. (Related to Figure 6.)

Movie 10. N-Cadherin Is Required for Contact Inhibition of Locomotion in Invasion and Collision Assays Invasion assays. Control NC explants fail to invade each other (top left) while explants treated with N-Cadherin blocking antibody are invading each other (top right). One picture every 5 min. Collision assays. Control cells change direction upon each collisions showing clear contact inhibition of locomotion (bottom left) while cells treated with N-Cadherin blocking antibody ignore other cells around them (bottom right) and keep moving following a linear path. One picture every 3 min. For both assays only green cells were pre-treated with NCD2 antibody. (Related to Figure 7.)
