# Supplementary material for: Collective Chemotaxis Requires Contact-Dependent Cell Polarity
Source: Dev Cell. 2010 Jul 20;19(1):39–53. doi: 10.1016/j.devcel.2010.06.012 (PMC2913244; doi:10.1016/j.devcel.2010.06.012)
Supplement: Document S1. Four Figures [file mmc1.pdf]

Developmental Cell ***19***

Supplemental Information

Collective Chemotaxis Requires

Contact-Dependent Cell Polarity

Eric Theveneau, Lorena Marchant, Sei Kuriyama, Mazhar Gull, Barbara Moepps, Maddy Parsons, and Roberto Mayor

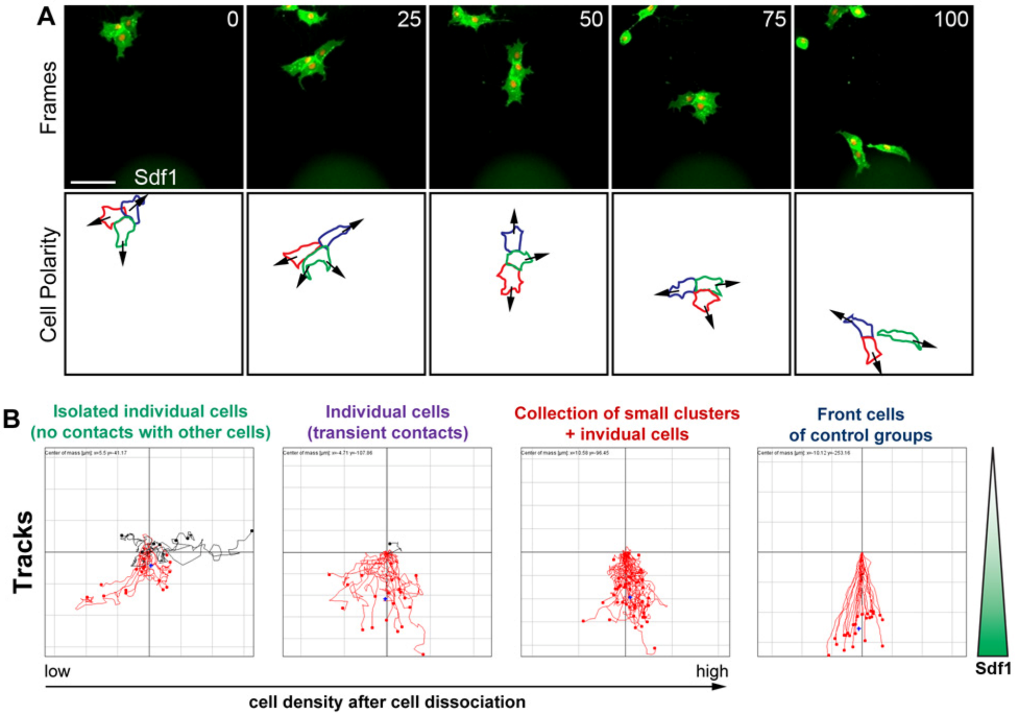

Figure S1, related to Figure 2. Cell interactions are essential for NC cells chemotaxis
(A) Migration of an isolated small cluster towards Sdf1. Despite having a good directionality at the cluster level each cell fail to properly orientate its cell body along the gradient reinforcing the idea that cell polarity is primarily established by cell contacts rather than by the chemokine. Time is minutes. Scale bar 100μm. (B) Tracks of isolated single cells (green), single cells with transient contacts (purple), single cells interacting with small clusters (red) and large clusters (blue). Chemotaxis improves as density increases; corresponding data are shown in Figure 2S-2T.

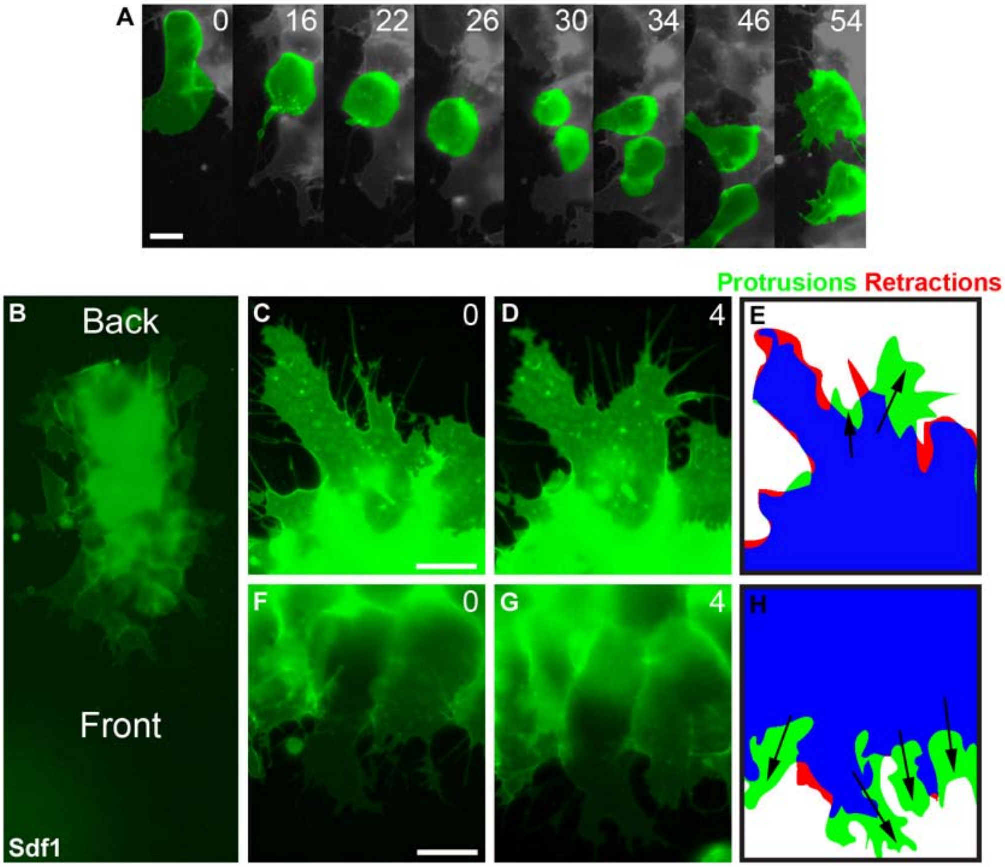

Figure S2, related to Figure 3. NC cells population contains non-responding cells
(A) A dividing cell shown here as an extreme example of non-motile cells being carried passively by motile cells.
(B-H) Comparison between front and back of an explant exposed to Sdf1 showing that both are producing protrusions. Cells at the back (C-E) and cells at the front (F-H) produce protrusions opposite to the cell contact region regardless of the orientation of the gradient. (E, H) Overlap of the two frames presented, steady part of the cell is in blue, protrusions are in green and retractions regions are in red. Time in minutes. Scale bars 10μm.

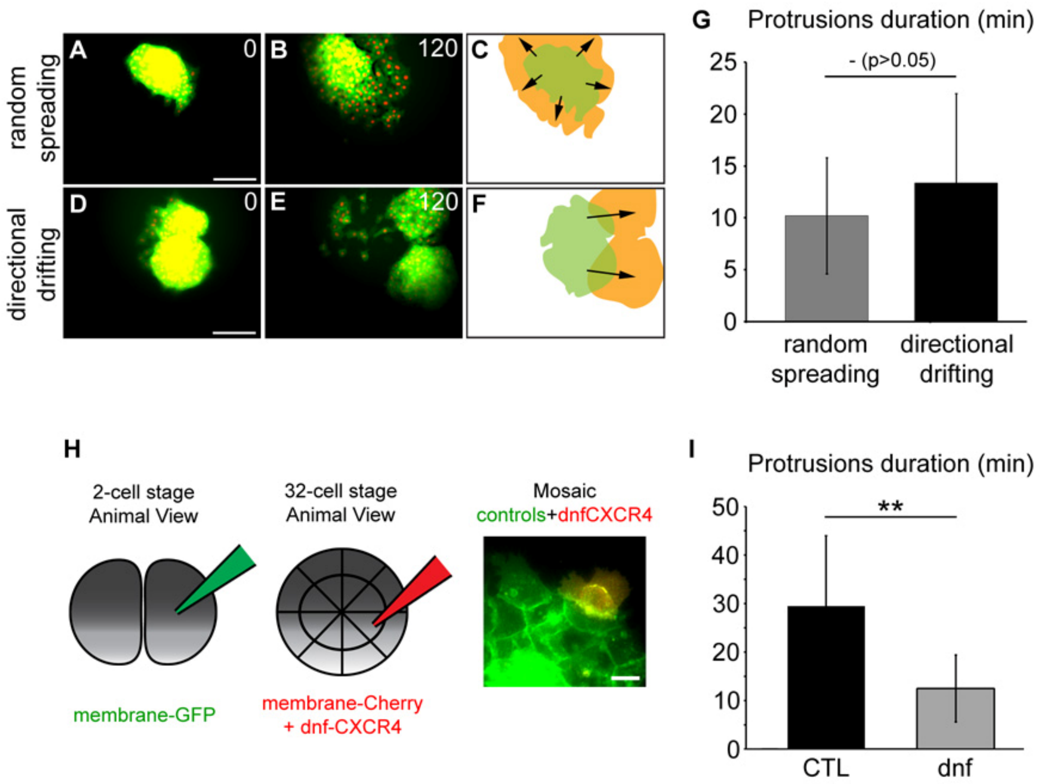

Figure S3, related to Figure 4. Protrusions stability depends on Sdf1 but not on cell coordination

(A-G). Analysis of cell protrusions stability in control explants spreading randomly (A-C, G, 10.2±5.6, n=37) or drifting directionally (D-F, G, 13.35±8.6, n=39) show no difference between the two conditions (p=0.067). (C, F) Global movement of each condition is shown by overlapping the two frames presented. Original position is in green, new location is in orange and cell movements are indicated by black arrows. (H-I) Control cells at the front of a control explant exposed to sdf1 show high protrusion stability (29.45±14.45, n=11) while dnf-Cxcr4 cells show a low protrusions stability (12.5±6.91, n=24, p=0.003). No difference was observed in protrusion stability in absence of Sdf1 (control cells: 11.2±6.8; dnCxcr4 cells: 14.2±7.8; n=15 for each conditions, p=0.268). Time in minutes. Scale bars 150µm (A-F), 10µm (H). Error bars show standard deviation.

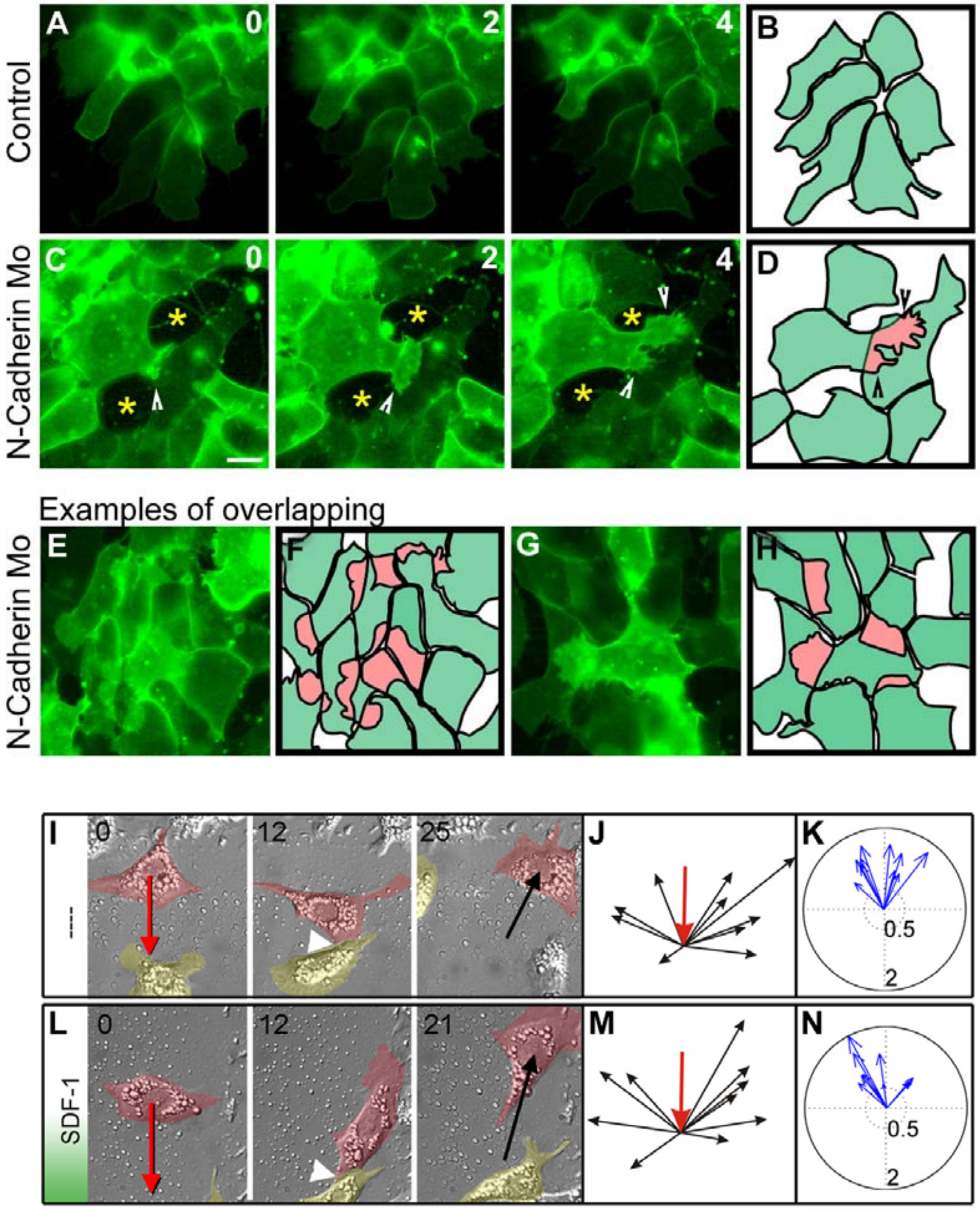

Figure S4, related to Figures 6. Contact-inhibition depends on N-Cadherin but is not influenced by Sdf1

(A-D) Frames of time lapse movies. Time in minute. (A-B) Control NC cells do not form protrusions in between each other. (C-D) N-Cadherin MO injected cells can form protrusions overlapping with neighbouring cells even if they had a previously established contact (C, arrowhead). This indicates that inhibition of N-Cadherin does not only promote cell dispersion but also reduces the ability of NC cells to sense each other as exemplified by the formation of protrusions over other cells. Panels on the right represent the last frame presented for each condition. Overlapping membranes are shown in pink. Scale bars 10μm. (E, H) Examples of overlapping protrusion in N-cadherin depleted cells. (I) Control condition showing two cells going away from each other after collision. (L) Chemotaxis assay in the presence of an Sdf1 gradient showing a non-affected contact inhibition of locomotion after collision. (J-K, M-N) Velocity vectors and acceleration vectors after cell collision in control and Sdf1 conditions are shown in B-C and E-F respectively; the red arrow indicates the initial velocity vector; the black arrow indicates the velocity vector after cell collision; the blue arrow indicates the acceleration vector; showing that they are clustered after the collision ($p < 0.005$, n=10 for each condition). Time in minutes.
