# Supplementary figures and images for: Collective Chemotaxis Requires Contact-Dependent Cell Polarity
Source: Dev Cell. 2010 Jul 20;19(1):39–53. doi: 10.1016/j.devcel.2010.06.012 (PMC2913244; doi:10.1016/j.devcel.2010.06.012)

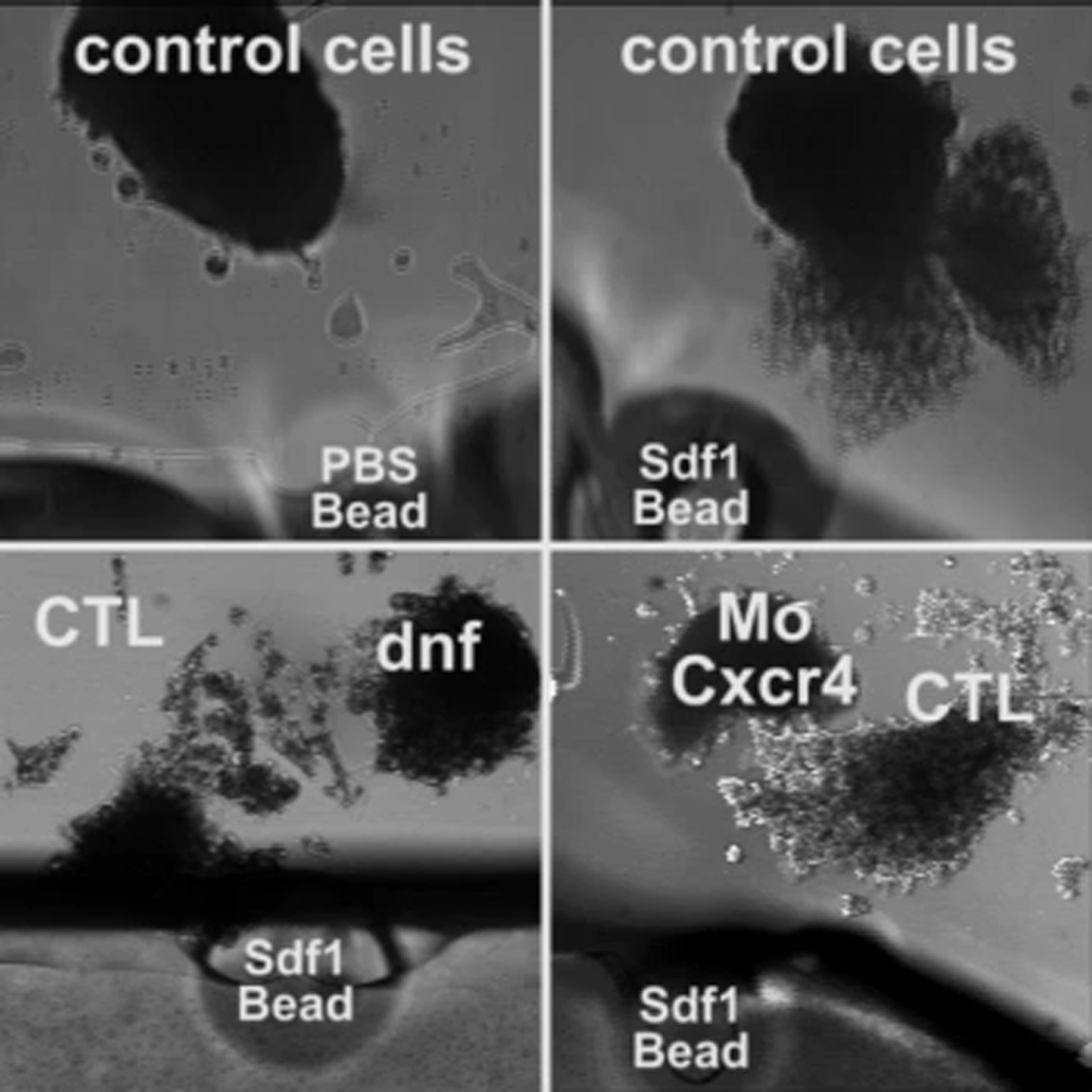

Supplement: Movie S1. Chemotaxis toward Sdf1 Requires Cxcr4 — Time-lapse movie of a fixed-bead chemotaxis assay with control NC cells confronted to PBS (top left) or Sdf1 bead (top right) and control NC cells competing with dnCxcr4 NC cells (bottom left) or Cxcr4-Mo NC cells (bottom right) in front of an Sdf1 bead. Only control NC cells exposed to Sdf1 are attracted. Without Sdf1 (top left) or when the response is inhibited by dnCxcr4 (bottom left) or Cxcr4-Mo (bottom right) no attraction is observed. One picture every 5 min. (Related to Figure 2.) [file mmc2.jpg]

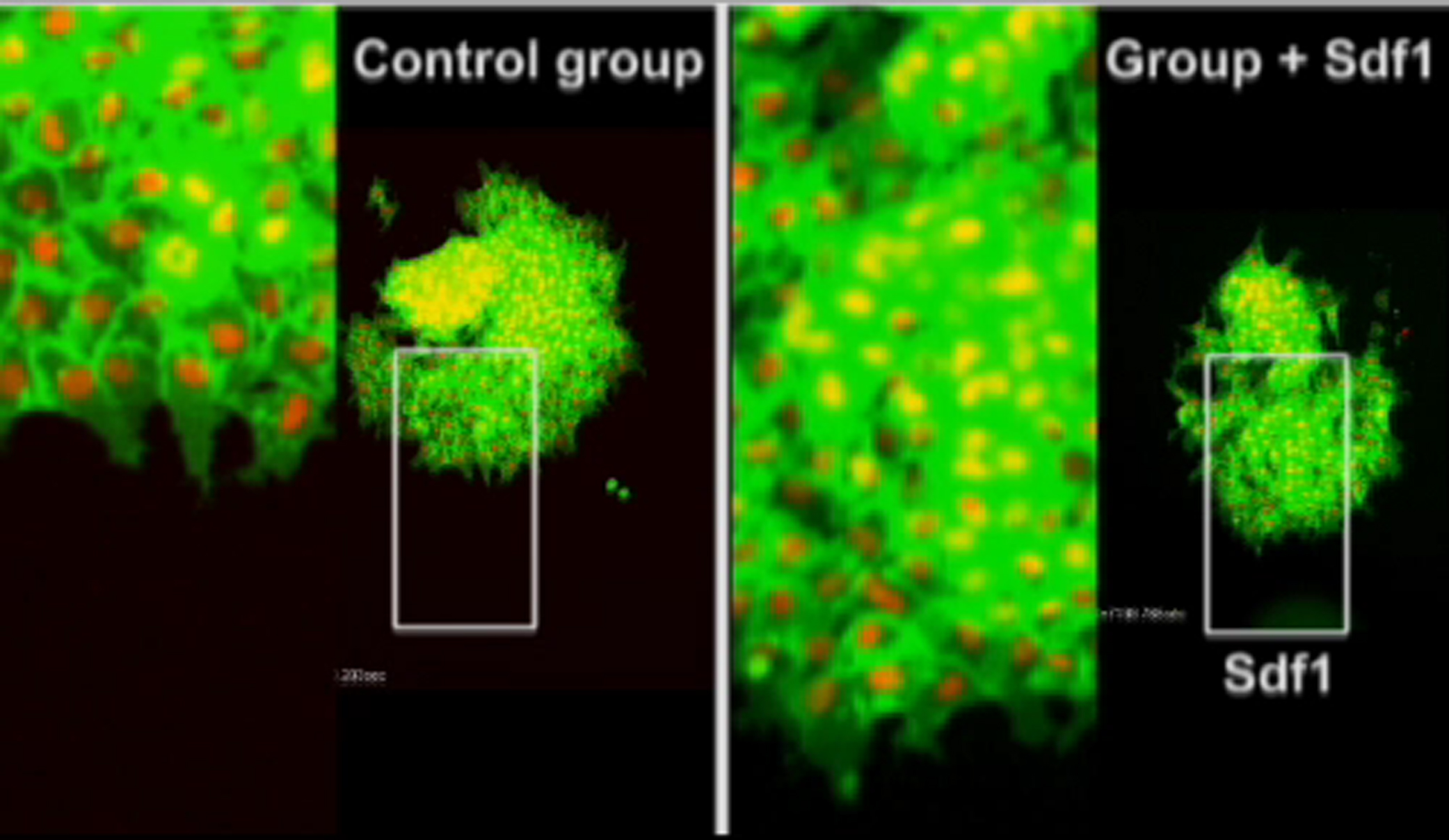

Supplement: Movie S2. Comparison between Control NC Cells Spreading and NC Cells Attracted to Sdf1 — Normal spreading of control NC cells (left) compared to control NC cells exposed to Sdf1 (right) in a fixed-bead chemotaxis assay. Cells were injected with nuclear-RFP and membrane GFP. Low magnification is shown to the right; rectangle indicates the higher magnification shown to the left. Cells exposed to Sdf1 undergo directional migration while control cells spread randomly from their original location. The net migration of the front when cells are exposed to Sdf1 is approximately 3× faster than when control cells spread randomly (110 μm/hr and 40 μm/hr, respectively). One picture every 5 min. (Related to Figure 2.) [file mmc3.jpg]

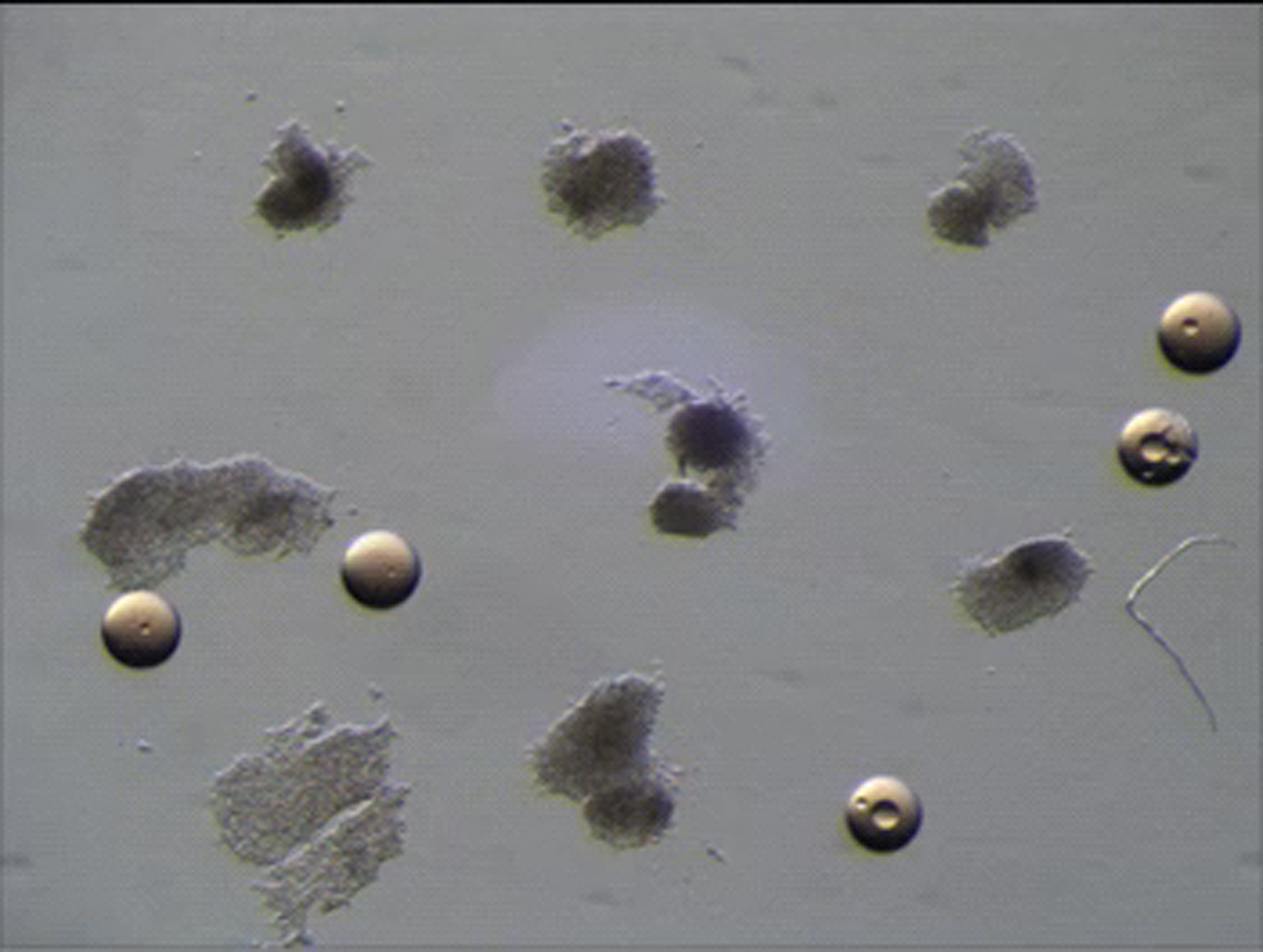

Supplement: Movie S3. Free-Bead Chemotaxis Assay with Sdf1 and PBS Beads — Free-bead chemotaxis assay with control NC cells exposed to PBS and Sdf1 beads. Sdf1 beads are on the left-hand side, PBS beads on the right-hand side. Note that NC cells actively track Sdf1 beads. When a bead moves to a new location it induces a change in cells direction of migration. On the contrary, PBS beads are ignored. One picture every 5 min. (Related to Figure 2.) [file mmc4.jpg]

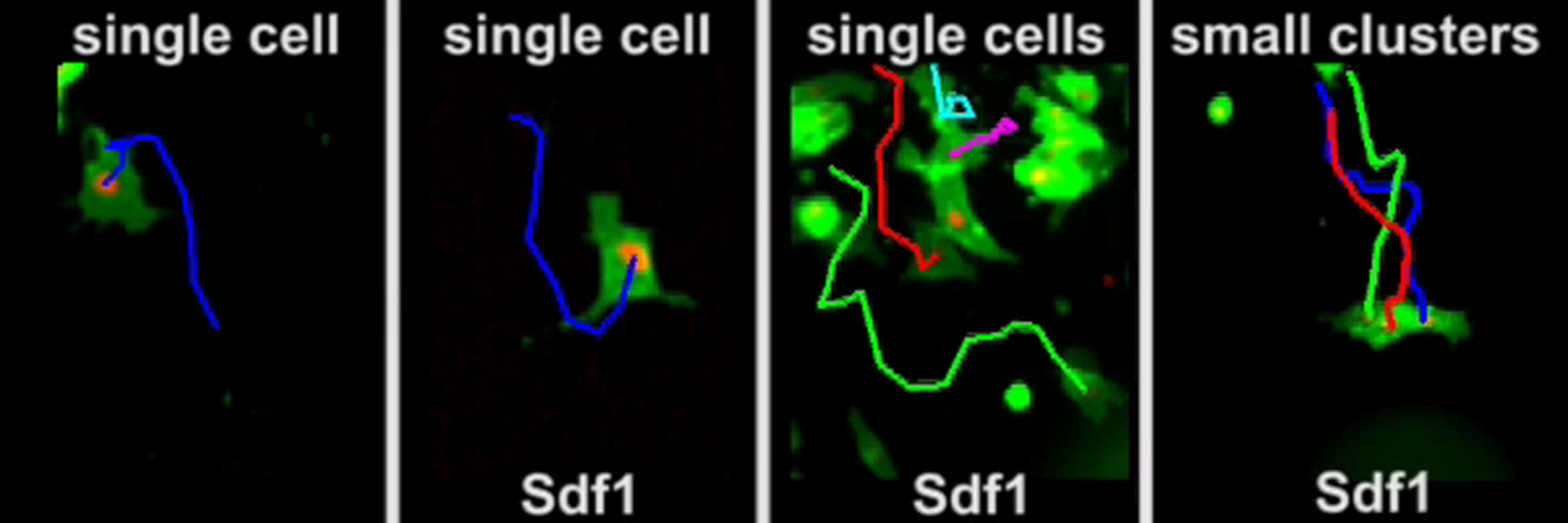

Supplement: Movie S4. Cell Contacts Are Required for Chemotaxis — Comparison between isolated single cells without Sdf1 (first panel) or exposed to Sdf1 (second panel), single cells with transient contacts exposed to Sdf1 (third panel) and isolated small clusters exposed to Sdf1 (fourth panel). These movies emphasize the fact that cell interactions, even transient, restore efficient chemotaxis (third and fourth panels) while the lack of cell contacts leads to inefficient chemotaxis (second panel). (Related to Figure 2.) [file mmc5.jpg]

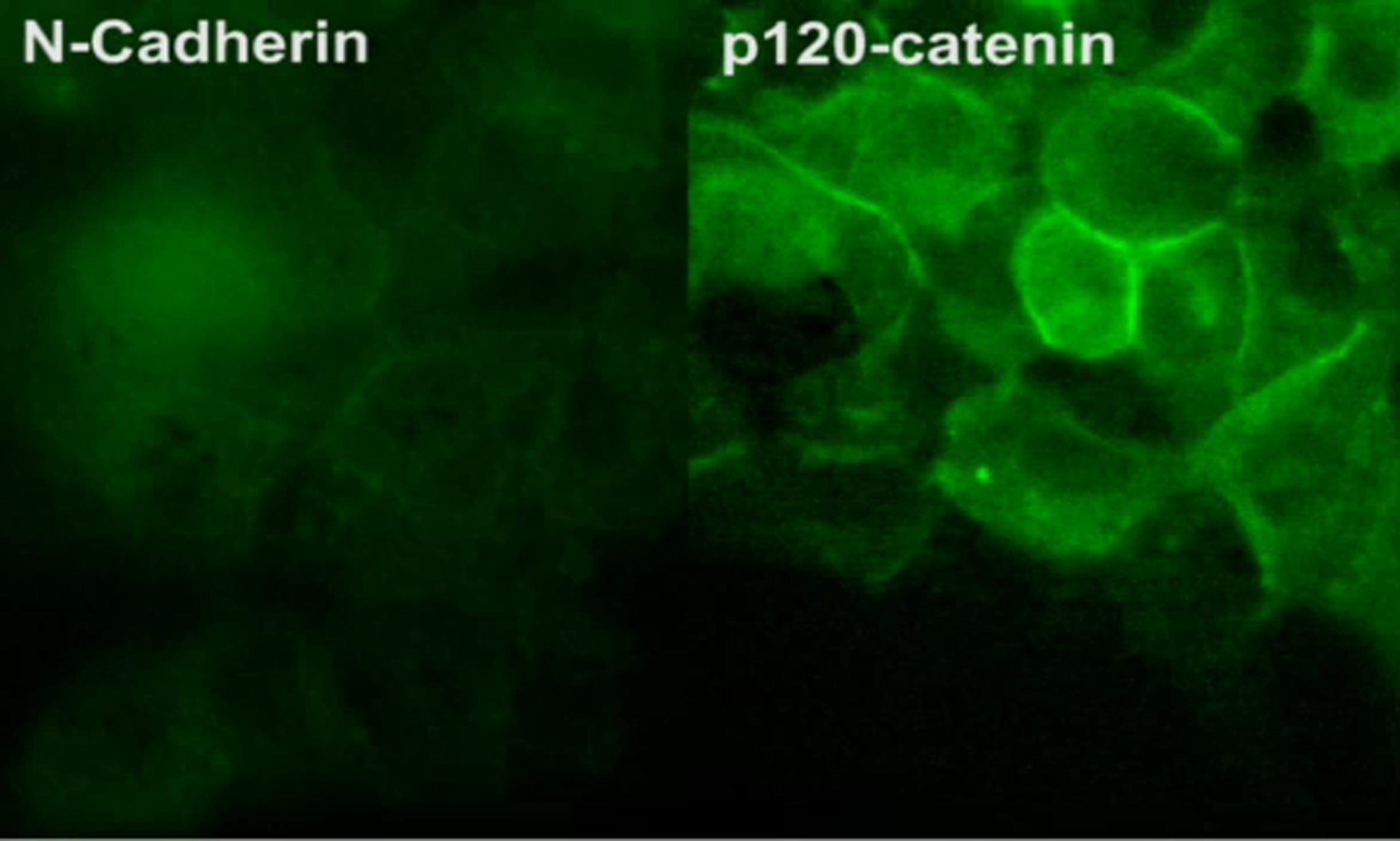

Supplement: Movie S7. N-Cadherin and p120 Localizations during NC Cells Migration — Membrane localization of N-cadherin-GFP during NC cells migration in vitro (left). Membrane localization of p120-GFP during NC cells migration in vitro (right). One picture every 90 s. (Related to Figure 5.) [file mmc8.jpg]
